# Uptake of COVID-19 vaccination among people who inject drugs

**DOI:** 10.1186/s12954-022-00643-3

**Published:** 2022-06-03

**Authors:** Jenny Iversen, Handan Wand, Robert Kemp, Jude Bevan, Myf Briggs, Kate Patten, Sue Heard, Lisa Maher

**Affiliations:** 1 The Kirby Institute, UNSW Sydney, Sydney, NSW 2052 Australia; 2 Queensland Health Department, Brisbane, Australia; 3 Western Australian Department of Health, Perth, Australia; 4 Tasmanian Department of Health, Hobart, Australia; 5 New South Wales Ministry of Health, Sydney, Australia

**Keywords:** People who inject drugs, COVID-19, SARS-CoV-2, Vaccine, Immunisation

## Abstract

**Background:**

People who inject drugs (PWID) may be at elevated risk of adverse outcomes from SARS-CoV-2 infection; however, data on COVID-19 vaccine uptake among PWID are scarce. This study aimed to determine COVID-19 vaccine uptake among PWID, identify factors associated with sub-optimal uptake, and compare uptake to the general population.

**Methods:**

The Australian Needle Syringe Program Survey is an annual sentinel surveillance project, comprising a self-completed questionnaire and provision of a dried blood sample for HIV and HCV testing. In 2021, respondents provided information on their COVID-19 vaccination status. Multivariate logistic regression models identified correlates of vaccine uptake.

**Results:**

Among 1166 respondents, 49% had been vaccinated and in most states and territories, vaccine uptake was significantly lower than among the general population. Independent predictors of vaccine uptake were longer duration of vaccine eligibility (AOR 3.42, 95% CI 2.65, 4.41); prior SARS-CoV-2 diagnostic testing (AOR 2.90, 95% CI 2.22, 3.79); injection of opioids (AOR 1.91, 95% CI 1.20, 3.05); and current opioid agonist therapy (AOR 1.70, 95% CI 1.23, 2.33). Women (AOR 0.70, 95% CI 0.54, 0.92) and those who reported daily or more frequent injection (AOR 0.75, 95% CI 0.57, 1.00) were significantly less likely to be vaccinated.

**Conclusions:**

In most Australian states and territories, uptake of COVID-19 vaccine among PWID lagged uptake among the general population. Increased efforts are required to ensure PWID have equitable access to vaccination. Vaccination programmes within harm reduction services and via outreach, coupled with increased support for peers to act as vaccine champions, are likely to reduce barriers and improve COVID-19 vaccine uptake in this population.

## Introduction

Severe acute respiratory syndrome coronavirus 2 (SARS-CoV-2), the novel virus that causes coronavirus disease (COVID-19), first emerged in late 2019. In March 2020, the World Health Organisation (WHO) declared SARS-CoV-2 a global pandemic, prompting the rapid and unprecedented development of safe and effective vaccines to prevent severe COVID-19 and reduce mortality [1]. By March 2022, there were 450 million confirmed cases of infection and six million deaths and 11 billion vaccine doses had been administered globally [2].

One of the key principles of the Australian COVID-19 Vaccination Policy [3] is to ensure the availability of COVID-19 vaccination for all Australians, at no cost to the consumer. However, given initial vaccine supply constraints, a phased national rollout of priority groups was outlined, with eligibility based on risk of exposure, priority workforces critical to the functioning of society, and risk of adverse health outcomes, including older people, those with underlying medical conditions, and Aboriginal and Torres Strait Islander people. As initial supply chain issues were ameliorated and vaccine eligibility relaxed, locations with capacity to administer vaccine were bought to scale, moving from hospital-based hubs and newly established respiratory clinics to general practitioners, community pharmacies, and special-purpose vaccination facilities such as state-run mass vaccination centres. On 30 August 2021, all Australians aged 16 years and over became eligible for vaccination, with eligibility extended to people aged 12–15 years in September 2021. Notwithstanding eligibility, administration of a vaccine dose generally required an individual to navigate the health system to book an appointment, typically using a complex web-based booking system [4]. In order to increase vaccine uptake, particularly in locations with COVID-19 outbreaks, walk in and pop-up clinics (no appointment required) were implemented in a range of settings, along with the use of community leaders as vaccine champions to normalise uptake and overcome hesitancy [4].

People who inject drugs (PWID) may experience a range of underlying medical conditions, including respiratory, pulmonary, compromised immunity, or chronic liver disease, that place them at elevated risk of adverse outcomes from SARS-CoV-2 infection [5–9]. Further, people with substance use disorders have been found to be more likely to experience breakthrough SARS-CoV-2 infection following vaccination, due to high prevalence of comorbidities, as well as adverse socio-economic circumstances, such as housing instability and economic disadvantage [10].

Although the World Health Organization (WHO) has stated that equitable access to vaccination is the key to ending the pandemic, PWID have lower uptake of influenza [11] and hepatitis B [12] immunisation than the general population [13]. PWID are typically under-represented in primary health care [14], with access to COVID-19 vaccination likely to be constrained by concerns about stigma and discrimination [15], costs associated with receipt of a vaccine [16], including transportation [17], the complexity of booking systems [4], and a lack of trust in government, which has been demonstrated to be associated with vaccine hesitancy in this population [18]. This study examined COVID-19 vaccine uptake among a large sample of PWID attending Australian needle syringe programmes (NSPs) in late 2021 and identified factors associated with vaccine uptake among this group.

## Materials and methods

### Study population

The Australian NSP Survey (ANSPS) is a sentinel surveillance project designed to monitor injection drug use, injection risk behaviour, and prevalence of HIV and hepatitis C virus (HCV) among PWID. The cross-sectional survey, conducted annually in all states and territories in Australia since 1995, comprises a self-administered questionnaire and provision of a dried blood spot for HIV and HCV testing. Respondents were recruited at selected NSP services, where they provided consent for voluntary and anonymous participation. The ANSPS methods are described in detail elsewhere [19], and previous research has demonstrated that ANSPS samples are as representative of the broader population of PWID as practicable to obtain [20]. Ethical approval for the ANSPS was provided by the Sydney Local Health District Ethics Review Committee (RPAH Zone) under the National Mutual Acceptance scheme (2019/ETHO7546), and the Tasmanian Health and Medical Human Research Ethics Committee (H0017666), with site-specific and external entity approvals obtained for all participating sites.

### Study outcome

In 2021, the ANSPS included a module to assess the impacts of the COVID-19 pandemic, including a question on COVID-19 vaccine uptake. Specifically, respondents were asked if they had been vaccinated for COVID-19, with the following available responses: (a) Yes, one dose, (b) Yes, two doses, and (c) No doses. A follow-up question was “If yes, what was the vaccine?”, with available responses: (a) AstraZeneca, (b) Pfizer, and (c) Don’t know. As Australia was experiencing COVID-19 surges in 2021, a 2-week ANSPS implementation was staggered over different time periods nationally. Queensland implemented the ANSPS in mid-September; Northern Territory, Tasmania, and Western Australia implemented in mid-October; South Australia commenced implementation in late November; and the Australian Capital Territory, New South Wales, and Victoria implemented the ANSPS in December. At the time of the 2021 ANSPS implementation, two vaccines (Vaxzevria^™^ developed by AstraZeneca/University of Oxford and Comirnaty^™^ developed by BioNTech/Pfizer) were available in Australia, with both vaccines commonly referred to by the name of the manufacturer. As per manufacturer recommended schedules and 2021 advice from the Australian Technical Advisory Group (ATAGI), at the time of 2021 ANSPS implementation, one dose of either vaccine was considered partial vaccination, while two doses were considered full vaccination [21].

### Statistical analysis

Vaccinated PWID (one or two doses) were assessed as the proportion of total respondents after excluding those who did not answer the question on vaccine uptake and respondents where the date of vaccine eligibility could not be determined. Data on the proportion of the general population who had received one or two doses of vaccine (in each state and territory on the date that ANSPS implementation commenced) were obtained from the daily COVID-19 vaccination rollout updates provided by the Australian Government Department of Health [22]. Given the phased nature of Australia’s vaccine rollout, which resulted in people becoming eligible for vaccination at different time points depending on their age, employment, underlying medical conditions, and Aboriginal and Torres Strait Islander background, a new variable was generated to determine duration of vaccine eligibility for each respondent. The number of days (divided by 30.5 to convert into months) respondents were eligible for vaccination was created by taking (a) the date of survey completion (using the first date of ANSPS implementation in each jurisdiction) minus (b) the date each respondent became eligible for vaccination based on their age, Aboriginal and Torres Strait Island background, HIV serostatus, and the state or territory of residence, noting that most states and territories implemented phased vaccine rollout that varied from the national timeline. It should also be noted that employment status and underlying medical conditions, other than HIV, may have resulted in vaccine eligibility at an earlier date, but these data were not collected. For this purpose, exposure to HCV was not included as an underlying medical condition as many people exposed to HCV clear the virus spontaneously or have cleared through treatment [23] and exposure to HCV has limited utility as a proxy for chronic liver disease.

Bivariate associations (*p* < 0.05) were examined using the Pearson’s chi-square test. Logistic regression models were used to estimate crude and adjusted odds ratios (AOR) and 95% confidence intervals (95% CI) to identify factors associated with COVID-19 vaccine uptake (one or two doses). Factors hypothesised to be associated with vaccine uptake among PWID were assessed. Demographic characteristics included in the model were gender [24], sexual identity [25], age [26], Aboriginal and Torres Strait Island background [27], and language spoken at home by parents [4]. The following variables were also hypothesised to be associated with vaccine uptake: HIV and HCV antibody serostatus [28, 29], frequency of injection and drug last injected as a proxies for severity of substance use disorder and possible indicators of structural barriers to accessing health care [30], and current opioid agonist therapy (OAT) and recent (past year) imprisonment given the potential for greater access to health professionals to facilitate vaccination in these settings. A history of COVID-19 testing [31] was also included in the model, as people who had a reason to test for COVID-19 may have had more incentive and/or greater access to vaccination. Duration of vaccine eligibility was also included in the model; however, age was excluded due to confounding. All variables associated with the outcome at *p* < 0.10 in bivariate analyses were considered in multivariable logistic regression models using a backwards stepwise approach. Factors were sequentially eliminated according to the result of a likelihood ratio test. All analyses were conducted using STATA software version 14.2 (Stata Corporation, College Station, TX, USA).

## Results

### Sample characteristics

Among 1474 ANSPS respondents in 2021, *n* = 240 (16%) did not answer the COVID-19 vaccine question and were excluded. A further 68 respondents (5%) were excluded who did not report whether they had an Aboriginal and Torres Strait Island background or not (*n* = 61) or report their age (*n* = 7), and the date of vaccine eligibility could not be determined. There was no significant difference in the COVID-19 vaccination status of respondents excluded because date of vaccine eligibility could not be determined and those retained in the dataset (*p* = 0.616). Among the 1166 respondents retained, almost two-thirds (62%) were male, the majority (81%) identified as heterosexual, the median age was 44 years (interquartile range [IQR] 37–50 years), and one in four (25%) identified as Aboriginal and Torres Strait Islander people (Table 1). A minority (3%) of respondents reported that their parents spoke a language other than English at home. Half of respondents (51%) reported last injecting methamphetamine, while 39% reported last injecting an opioid, including 22% who reported last injecting heroin. Ten per cent of respondents (*n* = 111) reported injecting other drugs at their last injection, including injection of multiple drugs (*n* = 61), cocaine (*n* = 6), and performance- and image-enhancing drugs (*n* = 34). One in ten respondents (12%) reported they had not injected in the previous month, while the remaining respondents were evenly distributed between those who injected daily or more frequently (44%) and those who injected less than daily (42%). One in four respondents (26%) reported they were currently receiving OAT, one in ten (10%) had been in prison in the last 12 months, and one in three (36%) reported having had a diagnostic test for SARS-CoV-2 infection. One in three (34%) respondents had been exposed to HCV, while a minority (1.6%) were living with HIV. Among those who had been tested for SARS-CoV-2 infection, a minority (*n* = 16, 4%) self-reported a positive result, equating to an overall self-reported SARS-CoV-2 prevalence of 1%.

**Table 1:** Number of COVID-19 vaccine doses received among ANSPS respondents in 2021

Characteristics	Total sample (*n* = 1166)	No doses (*n* = 598)	One dose (*n* = 219)	Two doses (*n* = 349)
Gender				
Male	726 (62)	352 (48)	147 (20)	227 (31)
Female	422 (36)	236 (56)	70 (17)	116 (27)
Sexual orientation				
Heterosexual	946 (81)	485 (51)	181 (19)	280 (30)
Bisexual	127 (11)	71 (56)	21 (17)	35 (28)
Gay/lesbian	56 (5)	20 (36)	8 (14)	28 (50)
Age				
< 40 years	393 (34)	243 (62)	58 (15)	92 (23)
40–49 years	429 (37)	229 (53)	86 (20)	114 (27)
50+ year	344 (30)	126 (37)	75 (22)	143 (42)
Aboriginal and Torres Strait Islander				
No	870 (75)	447 (51)	161 (19)	262 (30)
Yes	296 (25)	151 (51)	58 (20)	87 (29)
Parents language at home				
Not English	40 (3)	20 (50)	8 (20)	12 (30)
English	1118 (96)	574 (51)	209 (19)	335 (30)
Frequency of injection				
Less than daily	487 (42)	236 (48)	105 (22)	146 (30)
Daily	517 (44)	282 (55)	87 (17)	148 (29)
No injection last month	147 (13)	68 (46)	25 (17)	54 (37)
Drug last injected				
Other drugs	111 (10)	69 (62)	17 (15)	25 (23)
Methamphetamine	599 (51)	323 (54)	109 (18)	167 (28)
Heroin/other opioids	452 (39)	202 (44)	93 (21)	157 (35)
Current OAT				
No	862 (74)	479 (56)	153 (18)	230 (27)
Yes	304 (26)	119 (39)	66 (22)	119 (39)
Imprisonment				
No	1013 (87)	516 (51)	189 (19)	308 (30)
Yes	120 (10)	60 (50)	25 (21)	35 (29)
SARS-CoV-2 diagnostic test				
No	747 (64)	457 (61)	122 (16)	168 (22)
Yes	419 (36)	141 (34)	97 (23)	181 (43)
HIV status				
Negative	1112 (95)	577 (52)	209 (19)	326 (29)
Positive	19 (2)	3 (16)	4 (21)	12 (63)
Hepatitis C antibody status				
Negative	738 (63)	371 (50)	132 (18)	235 (32)
Positive	393 (34)	207 (53)	81 (21)	105 (27)
Duration of eligibility				
≤ 4.0 months	604 (52)	403 (66)	106 (18)	95 (16)
> 4.0 months	562 (48)	195 (35)	113 (20)	254 (45)

### COVID-19 vaccine uptake

All respondents were eligible for COVID-19 vaccination at the time of the study. The median duration of vaccine eligibility was 4.0 months (123 days of eligibility, IQR 97–161 days). Just over half of respondents (51%) had not been vaccinated, while 19% of respondents reported receiving one dose and 30% reported receiving two doses. Among those who had been vaccinated, half (52%) reported receiving Comirnaty^™^ (Pfizer) and 23% reported receiving Vaxzevria^™^ (AstraZeneca), while the remaining 25% either did not know which vaccine they had received (4%) or did not answer the question on the type of vaccine received (21%). Among those who reported the vaccine type, people who had received Comirnaty^™^ were more likely to have received two doses than those who reported receiving Vaxzevria^™^ (65% vs. 54%, respectively, *p* = 0.036).

Table 2 shows the proportion of PWID who had received any (one or two doses) COVID-19 vaccine compared to uptake among the general population eligible for vaccination in each state and territory on the first day of ANSPS implementation. COVID-19 vaccination among PWID (one or two doses) was significantly lower than among the general population in all states and territories, except the Northern Territory and New South Wales, noting that the New South Wales and Victorian samples were atypically small due to COVID-19 outbreaks that prevented several sentinel sites from participating in the ANSPS in 2021.

**Table 2:** COVID-19 vaccine uptake (%) among ANSPS respondents and among the eligible general population in Australia on the date the 2021 ANSPS commenced, by state and territory

State/territory	ANSPS commencement date	ANSPS respondents				Eligible general population^#^	
		*N*	Median days eligible (IQR)	Per cent 1st dose % (95% CI)	Per cent 2nd dose % (95% CI)	Per cent 1st dose	Per cent 2nd dose
Queensland	13 Sept 2021	331	97 (14–133)	30 (25, 35)	14 (11, 18)	57	39
Northern Territory	11 Oct 2021	36	133 (133–161)	64 (46, 79)	50 (33, 67)	69	57
Tasmania		76	133 (88–161)	58 (46, 69)	38 (27, 50)	81	65
Western Australia		389	123 (123–123)	43 (38, 48)	23 (19, 27)	71	53
South Australia	1 Nov 2021	170	146 (146–182)	52 (44, 59)	28 (21, 34)	82	67
Australian Capital Territory	7 Dec 2021	91	187 (126–218)	87 (78, 93)	76 (66, 84)	> 95	> 95%
New South Wales		35	204 (204–218)	100 (–, –)	77 (60, 90)	95	93
Victoria	13 Dec 2021	38	199 (199–224)	82 (66, 92)	58 (41, 74)	94	92

^#^COVID-19 vaccination rollout updates provided by the Australian Government Department of Health

In unadjusted analysis (Table 3), factors significantly associated with COVID-19 vaccine uptake were gender, age, HIV positive serostatus, drug last injected, current engagement with OAT, a prior diagnostic test for SARS-CoV-2 infection, and duration of vaccine eligibility. In adjusted analysis, longer duration of vaccine eligibility (adjusted odds ratio [AOR] 3.42, 95% confidence interval [CI] 2.65, 4.41, *p* < 0.001) and having had a prior SARS-CoV-2 diagnostic test (AOR 2.90, 95% CI 2.22, 3.79, *p* < 0.001) were the strongest predictors of vaccine uptake. People who last injected opioids (AOR 1.91, 95% CI 1.20, 3.05, *p* = 0.007) were significantly more likely to have been vaccinated than those who injected multiple or other drugs, while people currently receiving OAT were also significantly more likely to have been vaccinated than those not currently on OAT (AOR 1.70, 95% CI 1.23, 2.33, *p* = 0.001). Conversely, women were significantly less likely to have been vaccinated compared to men (AOR 0.70, 95% CI 0.54, 0.92, *p* = 0.009) and people who injected daily or more frequently were also significantly less likely to have been vaccinated compared to those who injected less than daily (AOR 0.75, 95% CI 0.57, 1.00, *p* = 0.042).

**Table 3:** Factors associated with COVID-19 vaccine uptake among ANSPS respondents in 2021

Characteristics	Univariable analysis		Multivariable analysis	
	Unadjusted odds ratio (95% CI)	*p* value	Adjusted^§^ odds ratio (95% CI)	*p* value
Gender				
Male	1		**1**	
Female	0.75 (0.59, 0.95)	0.017	**0.70 (0.54, 0.92)**	**0.009**
Sexual orientation				
Heterosexual	1		1	
Bisexual	0.82 (0.57, 1.120)	0.310	0.88 (0.58, 1.34)	0.543
Gay/lesbian	1.19 (0.77, 1.84)	0.421	1.16 (0.72, 1.87)	0.532
Age				
< 40 years	1		1	
40–49 years	1.41 (1.07, 1.87)	0.014	1.18 (0.87, 1.61)	0.295
50+ year	2.80 (2.07, 3.78)	< 0.001	1.12 (0.75, 1.67)	0.592
Aboriginal and Torres Strait Islander				
No	1		1	
Yes	1.01 (0.78, 1.32)	0.913	1.10 (0.81, 1.46)	0.586
Parents language at home				
Not English	1		1	
English	0.95 (0.53, 1.69)	0.856	0.87 (0.46. 1.64)	0.670
Frequency of injection				
Less than daily	1		1	
Daily	0.78 (0.61, 1.00)	0.054	**0.75 (0.57, 1.00)**	**0.042**
No injection last month	0.96 (0.68, 1.38)	0.839	**1.01 (0.68, 1.50)**	**0.972**
Drug last injected				
Other drugs (ref)	1		**1**	
Methamphetamine	1.49 (0.98, 2.24)	0.060	**1.57 (1.00, 2.50)**	**0.052**
Heroin/other opioids	2.15 (1.41, 3.28)	< 0.001	**1.91 (1.20, 3.05)**	**0.007**
Current OAT				
No	1		**1**	
Yes	1.94 (1.49, 2.54)	< 0.001	**1.70 (1.23, 2.33)**	**0.001**
Imprisonment				
No	1		1	
Yes	1.06 (0.73, 1.55)	0.766	1.34 (0.88, 2.05)	0.172
SARS-CoV-2 diagnostic test				
No	1		**1**	
Yes	3.11 (2.42, 4.00)	< 0.001	**2.90 (2.22, 3.79)**	**< 0.001**
HIV status				
Negative	1		1	
Positive	5.75 (1.67, 19.85)	0.006	2.96 (0.83, 10.52)	0.100
Hepatitis C antibody status				
Negative	1		1	
Positive	0.91 (0.71, 1.16)	0.442	1.01 (0.76, 1.33)	0.967
Duration of eligibility				
≤ 4.0 months	1		**1**	
> 4.0 months	3.77 (2.96, 4.81)	< 0.001	**3.42 (2.65, 4.41)**	**< 0.001**

^§^Bolded odds ratios are from multivariable model which included six variables: gender, frequency of injection, drug last injected, current OAT, SARS-CoV-2 test, and duration of vaccine eligibility; all other odds ratios were adjusted for these six variables

## Discussion

Less than half our community sample of PWID had received at least one dose of COVID-19 vaccine, with COVID-19 vaccination significantly lower among PWID than among the general community in most Australian states and territories. Vaccine hesitancy among PWID has been documented, with fear of side effects and vaccine safety concerns frequently reported [11, 32, 33]. Nonetheless, it is important to distinguish between vaccine hesitancy and barriers to access [4]. While only one in two Australian PWID in our study had been vaccinated, uptake was higher than elsewhere, with only 9% of PWID having received at least one dose of COVID-19 vaccine in the US–Mexico border region by September 2021 [29]. However, unlike Australia, where only 1% of our sample self-reported having had SARS-CoV-2 infection, in the Strathdee et al. (2021) study of 386 PWID, SARS-CoV-2 seroprevalence was 36% and people with recent infection may have delayed vaccination in that setting. No other reports of vaccine uptake among PWID were identified in the literature globally, suggesting that this sub-population has been largely overlooked [7, 34].

Duration of vaccine eligibility and having had a diagnostic test for SARS-CoV-2 infection were associated with higher vaccine uptake. This suggests that PWID may be willing to be vaccinated given sufficient opportunity to do so, with vaccine appointments generally becoming more accessible over time as supply chain issues were addressed, wait lists declined, and walk-in, outreach, and pop-up clinics (where no appointment was required) were established. It also appears that PWID sufficiently concerned about possible exposure to warrant diagnostic testing may be more motivated to be vaccinated. This finding was also observed in the US–Mexico border region, where a similar proportion of PWID (32%) had received diagnostic testing for SARS-CoV-2 [29] and testing was associated with higher vaccine uptake [31]. Further, in a multicity comparative study conducted in the general population in the USA, UK, and Australia, knowing someone personally who had COVID-19 was positively associated with willingness to be vaccinated [18].

In our study, women who inject drugs were less likely to be vaccinated than their male counterparts. This is consistent with the literature on hypothetical vaccine hesitancy among the general population. Kaufman et al. (2021) reported that Australian women were less likely to accept a COVID-19 vaccine than men and that young women in particular reported concerns around pregnancy, breastfeeding, and fertility specific to the COVID-19 vaccine. In another Australian study of attitudes towards vaccines and immunisation intent in the general population, there were no gender differences in vaccine intentions for one’s self or children in general (for example routine childhood vaccinations), but men indicated significantly higher intent to obtain the COVID-19 vaccine than women [35].

The finding that people who reported receiving Comirnaty^™^ were more likely to have received two doses of vaccine than those who reported receiving Vaxzevria^™^ was expected and likely due to the shorter duration vaccine schedule for Comirnaty^™^ (3–6 weeks between dose one and two compared to 8–12 weeks for Vaxzevria^™^). Similarly, it was not unexpected that people currently engaged in OAT were significantly more likely to be vaccinated, likely due to health professionals providing accurate information, cautioning PWID about risks associated with severe COVID-19 disease, and facilitating access to vaccination in this setting [5]. Despite high uptake of vaccination (84%) among people living with HIV, and a large effect in univariable analysis (OR 5.75, 95% CI 1.67–19.85), HIV status was limited by the small sample size and wide confidence intervals and was not significant in the multivariable model. PWID with a history of recent imprisonment had comparable vaccine uptake to their non-incarcerated counterparts. This finding suggests that vaccine uptake among recently incarcerated PWID also lagged behind the general community at the time of 2021 ANSPS implementation. While this is of concern given the risk for clusters of SARS-CoV-2 infection to occur among people living in closed settings [36], Australian state and territory correctional facilities have implemented onsite COVID-19 vaccine programmes to support uptake among prison populations.

Although a lack of trust in government has been demonstrated to be associated with vaccine hesitancy, including in Australia [18], PWID typically have trusted relationships with harm reduction staff and peer-based organisations [37]. In the central Sydney area of New South Wales, where peer-based and government harm reduction services operated drop-in and/or outreach vaccination programmes for PWID and other key populations in the 6 months prior to implementation of the 2021 ANSPS, vaccine uptake in this small subsample was 100%, and three quarters of PWID had received two doses of vaccine. These results demonstrate that high vaccine uptake among PWID, at levels commensurate with uptake among the general population, can be achieved with concerted efforts, engagement of the peer-based workforce, and targeted outreach. Drop-in and outreach vaccination programmes have subsequently been established at harm reduction services, including peer-based services, in other Australian states and territories. These services, that aim to decrease barriers to vaccine uptake among PWID by reducing the potential for stigma and discrimination, removing additional transportation difficulties or costs, as well as bypassing appointment booking systems, are necessary to redress the lower vaccine uptake among PWID, particularly women and those who inject on a daily or more frequent basis and those with limited contact with health professionals.

Our study has some limitations. First, it is acknowledged that some people may have been eligible for vaccination at an earlier date due to employment as a critical worker or underlying medical conditions which we did not collect data on. Further, we were unable to determine vaccination status for all respondents. If the group with missing data avoided the COVID-19 vaccine question because they were unwilling to disclose their unvaccinated status, our study may have overestimated vaccine uptake among PWID. Although the ANSPS recruited respondents from all states and territories, the sample size was small in jurisdictions experiencing surges in COVID-19 infection in late 2021, particularly New South Wales and Victoria. Further, respondents were recruited from NSP services during the COVID-19 pandemic and do not represent PWID who do not or did not attend an NSP in late 2021. Lastly, data were self-reported and may be subject to social desirability bias, although previous research has demonstrated acceptable reliability and validity of PWID self-report [38] and the self-completed nature of the ANSPS reduces social desirability bias [39].

## Conclusions

In most Australian states and territories, uptake of COVID-19 vaccine among PWID lags behind uptake among the general population. Concerted efforts are required to ensure PWID have access to vaccination, particularly women and those who inject daily or more frequently. Vaccination programmes within harm reduction services and via outreach, coupled with increased support for the peer-based workforce to act as vaccine champions, are strategies likely to reduce barriers and improve COVID-19 vaccine uptake among PWID.

## Data Availability

The data analysed during the current study are not publicly available as Ethical Approval for the Australian NSP Survey does have a provision for data sharing. Please contact the corresponding author with any reasonable requests.

## Abbreviations

ANSPS: Australian Needle Syringe Program Survey
AOR: Adjusted odds ratio
CI: Confidence interval
HCV: Hepatitis C virus
HIV: Human immunodeficiency virus
NSP: Needle syringe programme
OAT: Opioid agonist therapy
PWID: People who inject drugs
SARS-CoV-2: Severe acute respiratory syndrome coronavirus 2
WHO: World Health Organisation
